# Long-term follow up of fecundability after ectopic pregnancy in Taiwan: a nationwide population-based study

**DOI:** 10.3389/fmed.2024.1430570

**Published:** 2024-10-24

**Authors:** Chih-Hsiang Yin, Yi-Liang Lee, Chia-Ching Chang, Wu-Chien Chien, Gwo-Jang Wu

**Affiliations:** ^1^Department of Obstetrics and Gynecology, Tri-Service General Hospital, National Defense Medical Center, Taipei, Taiwan; ^2^National Defense Medical Center, Taipei, Taiwan; ^3^Department of Obstetrics and Gynecology, Kang Ning Hospital, Taipei, Taiwan; ^4^National Defense Medical Center, Graduate Institute of Life Sciences, Taipei, Taiwan; ^5^Department of Medical Research, National Defense Medical Center, Tri-Service General Hospital, Taipei, Taiwan; ^6^National Defense Medical Center, School of Public Health, Taipei, Taiwan; ^7^Taiwanese Injury Prevention and Safety Promotion Association, Taipei, Taiwan

**Keywords:** ectopic pregnancy, pregnancy, pregnancy rate, fecundability, national health insurance research database

## Abstract

**Background:**

Ectopic pregnancy (EP) occurs when a fertilized ovum is implanted outside the uterine cavity. Its incidence is 2% of all pregnancies and is known to decrease fertility. This study aimed to measure pregnancy rates after EP, identify the various parameters influencing pregnancy, and compare these variables in women with and without a history of EP, as well as determine in which medical facilities women with EP seek medical help.

**Methods:**

The data was extracted from the National Health Insurance Research Database during 2000–2013. The study group included of women with a history of one EP (study group) and women without EP (control group). The chi-square/Fisher exact test was performed for categorical variables, and t-tests were used for continuous variables.

**Results:**

The EP group had a higher cumulative pregnancy rate (41.55%) than the control group (37.14 %), and a 1.16 times higher rate in developing pregnancy (*p* < 0.001). While the pregnancy rate in the EP group was initially lower than in the control group during the first 5 years, it surpassed the control group’s rate between the 10*^th^* and 14*^th^* years.

**Conclusions:**

Fecundability after EP was lower at the beginning but increased at long-term follow-up.

## 1 Introduction

Ectopic pregnancy (EP) is a potentially life-threatening condition which defined as the implantation of a fertilized ovum outside the uterus, with 98% occurring in the fallopian tube in the first trimester (1). The diagnosis of EP relies on the clinical presentation of the patient, pregnancy test results, quantitative beta-human chorionic gonadotropin (β-hCG) blood levels, ultrasound visualization of the embryo in the adnexa outside of the uterine cavity, or even diagnostic laparoscopy. Early diagnosis is crucial because EP accounts for 5–10% of all pregnancy-related deaths. When EP ruptures, the maternal mortality rate increases to 9–14% (2). In addition to its implications for morbidity and mortality, EP leads to the deterioration of psychological health such as anxiety to the outcome of treatment and depression caused by pregnancy loss (2). The main risk factors for EP include tubal damage caused by surgery or genital infection, age, cigarette smoking, intrauterine device placement, previous EP, and infertility (3). Management of EP includes medical, surgical, or observation, largely depending on β-hCG levels, the EP’s location, gestational age, willingness to preserve fertility, and patient’s condition (2, 3).

The incidence of EP is estimated at 2% of all pregnancies (4). One study revealed that the majority of patients with EP were primigravidas (14/45, 31.3%) and had no apparent risk factors of EP (20/45, 44.44%) (5). Since EP is a common and serious condition affecting women of reproductive age with the possibility of subsequent infertility (6), more attention should be paid to patients with EP, especially in Taiwan, as the birth rate has rapidly declined below replacement levels. According to the Central Intelligence Agency of the United States, the crude birth rate in Taiwan will be 7.3 births per 1,000 people in 2023, ranking 9^th^ from the bottom globally (7).

Advances in medical diagnostics have shifted physicians’ focus from the immediate health consequences to fertility preservation in women with EP (8). However, there is a lack of comprehensive analyses of fertility rates after EP. To address this problem, we elucidated various parameters influencing pregnancy after EP through a population-based study by analyzing data from the Taiwan National Health Insurance Research Database (NHIRD), a powerful tool for studying the epidemiology of Taiwan. This study investigated pregnant conditions after EP and related variables such as age, economic condition, comorbidities, season or the year, residence area, urbanization level, and the healthcare facilities where women with EP seek medical help.

## 2 Materials and methods

### 2.1 Data extraction

Data were extracted from the NHIRD to determine the factors influencing the pregnancy rate after EP. The database was from the Taiwan’s National Health insurance (NHI) program, which was established in 1995. Insurance coverage of the Taiwanese population increased from 93% to 100% between 1996 and 2023 (9). The number of individuals included was sufficiently large to represent the population of Taiwan. Disease diagnosis was based on the International Classification of Diseases, Ninth Revision, Clinical Modification (ICD-9-CM).

### 2.2 Ethics statement

This study was conducted in accordance with the Ethics Code of the World Medical Association (Declaration of Helsinki). This study was approved by the Institutional Review Board (IRB No. C202405034) of the Tri-Service General Hospital. The requirement for patient consent was waived because the data were extracted from the NHIRD.

### 2.3 Study design

This retrospective cohort study included 989,753 female individuals recorded in the outpatient and inpatient databases between 2000 and 2013 in Taiwan. The study included 5,087 women with EP by searching ICD-9-CM codes 633.0–633.2 and 633.8–633.9. Exclusion criteria were as follows: EP before the index date, pregnancies that occurred before tracking, and women aged under 12 or over 50 years. After applying these criteria, 59 individuals were further excluded from the study.

**FIGURE 1:**
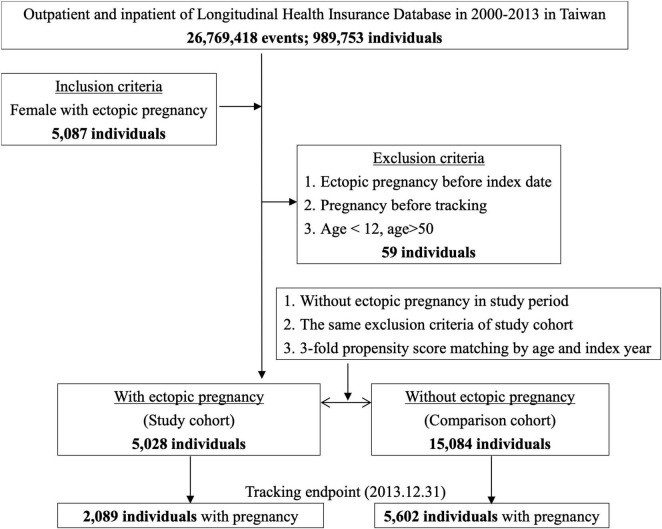
Flowchart of study sample selection from National Health Insurance Research Database in Taiwan.

The control group included women who visited a doctor during the same period, but without an EP. The same exclusion criteria were applied to both groups. This study employed propensity score matching to construct a control group by matching age and index year. Accordingly, the number of control group participants was set at maintaining a 1:3 ratio to the experimental group, resulting in a total of 15,084 individuals included in the study. A flowchart of the study sample selection is shown in Figure 1.

Pregnant was defined as having a positive pregnant test, seeking medical care during pregnancy, or both.

### 2.4 Statistical analysis

Statistical analyses were performed using SPSS software (SPSS Inc., Chicago, IL, USA). To further describe the details of the ectopic pregnant women and their control group, we listed various variables such as age, insurance premiums (NT$, New Taiwan Dollar), presence of complications such as anemia and shock, season of the year, residence area, urbanization level, and level of care. Insurance premiums indirectly reflect income. The higher the income, the higher the insurance premium paid. The chi-square/Fisher’s exact test was performed on categorical variables, and the t-test was used for continuous variables.

## 3 Results

Characteristics of the study population are summarized in Table 1. This study included 20,112 participants, comprising 5,028 patients with EP and 15,084 individuals without EP. Patients with EP had a higher cumulative incidence rate of pregnancy of 41.55% (2,089/5028 individuals) than the control group with 37.14% (5,602/15,084 individuals). The majority of the study population had an average age of 33.73 ± 8.12 years. There was a higher percentage of patients aged 30–39 years in the EP group than in the control group (49.6% vs. 43.27%, *p* < 0.001). Furthermore, patients with EP had a higher cumulative incidence of anemia than the control group (0.91% vs. 0.43%; *p* < 0.001). The incidence of shock was similar between the two groups. In addition, the number of visits to the physician was distributed equally among seasons (23.95% in spring, 25.7% in summer, 25.99% in autumn, and 24.36% in winter; *p* = 0.306). Patients in both groups predominantly resided in Northern Taiwan and the urbanization level 2 area of Taiwan. The EP group tended to seek treatment at local hospitals (40.27% vs. 36.79%, *p* < 0.001).

**TABLE 1:** Characteristics of the study.

	Total population		With ectopic pregnancy		Without ectopic pregnancy		*P-Value*
**Variables**	***n***	**%**	***n***	**%**	***n***	**%**	
**Total**	20,112		5,028	25.00	15,084	75.00	
**Pregnancy**							< 0.001
Without	12,421	61.76	2,939	58.45	9,482	62.86	
With	7,691	38.24	2,089	41.55	5,602	37.14	
**Age (years)**	33.73 ± 8.12		33.17 ± 7.09		33.91 ± 8.43		< 0.001
**Age group (years)**							< 0.001
12–19	434	2.16	95	1.89	339	2.25	
20–29	6,647	33.05	1,639	32.60	5,008	33.20	
30–39	9,021	44.85	2,494	49.60	6,527	43.27	
≧40	4,010	19.94	800	15.91	3,210	21.28	
**Insured premium (NT$)**							0.828
<18,000	18,000	89.50	4,498	89.46	13,502	89.51	
18,000–34,999	1,569	7.80	399	7.94	1,170	7.76	
≧35,000	543	2.70	131	2.61	412	2.73	
**Anemia**							< 0.001
Without	20,001	99.45	4,982	99.09	15,019	99.57	
With	111	0.55	46	0.91	65	0.43	
**Shock**							0.407
Without	20,016	99.52	5,008	99.60	15,008	99.50	
With	96	0.48	20	0.40	76	0.50	
**Season of the year**							0.306
Spring (March–May)	4,816	23.95	1,207	24.01	3,609	23.93	
Summer (June–August)	5,169	25.70	1,246	24.78	3,923	26.01	
Autumn (September–November)	5,227	25.99	1,315	26.15	3,912	25.93	
Winter (December–February)	4,900	24.36	1,260	25.06	3,640	24.13	
**Location**							0.019
Northern Taiwan	8,715	43.33	2,252	44.79	6,463	42.85	
Middle Taiwan	5,352	26.61	1,297	25.80	4,055	26.88	
Southern Taiwan	4,945	24.59	1,194	23.75	3,751	24.87	
Eastern Taiwan	1,029	5.12	274	5.45	755	5.01	
Outlets islands	71	0.35	11	0.22	60	0.40	
**Urbanization level**							< 0.001
1(The highest)	7,614	37.86	1,970	39.18	5,644	37.42	
2	8,558	42.55	2,180	43.36	6,378	42.28	
3	1,568	7.80	371	7.38	1,197	7.94	
4 (The lowest)	2,372	11.79	507	10.08	1,865	12.36	
**Level of care**							< 0.001
Hospital center	6,219	30.92	1,468	29.20	4,751	31.50	
Regional hospital	6,319	31.42	1,535	30.53	4,784	31.72	
Local hospital	7,574	37.66	2,025	40.27	5,549	36.79	

*P:* Chi-square/Fisher exact test on category variables and *t*-test on continue variables.

Cox regression analysis of intrauterine pregnancy factors was performed after adjusting for the variables listed in Table 2. The analysis revealed that patients with EP had a 1.16 times higher rate in developing pregnancy than patients without EP (*p* < 0.001). Individuals aged 20–29 years had a significantly higher pregnancy rate than those aged 12–19 years. Patients with higher income, who paid > 35,000 NT insurance premium per year, had a 1.62-fold higher pregnancy rate than those in the lower income group (*p* < 0.001). Anemia, shock, and season of the year did not affect pregnancy. A lower pregnancy rate was observed in patients living in Southern Taiwan (0.94; *p* = 0.035) than in those living in Northern Taiwan. Compared with that of people who lived in the lowest urbanization areas, a relatively higher incidence of pregnancy was observed in residents of the most prosperous city, urbanization level 1 (1.136; *p* = 0.001); however, patients with urbanization level 3 scored the lowest pregnancy rate (0.852; *p* = 0.002). Patients who visited a local hospital had higher pregnancy rates than those treated at hospital centers (0.715; *p* < 0.001) and regional hospitals (0.749; *p* < 0.001).

**TABLE 2:** Factors of pregnancy by using Cox regression.

Variables	Crude HR [95% CI]	*P-Value*	Adjusted HR [95% CI]	*P-Value*
**Ectopic pregnancy**				
Without	Reference		Reference	
With	1.265 [1.203–1.331]	<0.001	1.160 [1.103–1.220]	< 0.001
**Age group (years)**				
12–19	Reference		Reference	
20–29	1.662 [1.359–2.033]	<0.001	1.589 [1.299–1.945]	<0.001
30–39	0.597 [0.569–0.853]	<0.001	0.689 [0.563–0.844]	<0.001
≧40	0.017 [0.013–0.023]	<0.001	0.018 [0.014–0.024]	<0.001
**Insured premium (NT$)**				
< 18,000	Reference		Reference	
18,000–34,999	0.828 [0.650–1.055]	0.127	0.961 [0.820–1.126]	0.622
≧35,000	0.818 [0.609–1.083]	0.157	1.621 [1.272–2.067]	<0.001
**Anemia**				
Without	Reference		Reference	
With	0.982 [0.714–1.351]	0.912	0.973 [0.707–1.339]	0.866
**Shock**				
Without	Reference		Reference	
With	0.000	0.774	0.000	0.691
**Season**				
Spring	Reference		Reference	
Summer	0.966 [0.906–1.031]	0.301	0.974 [0.912–1.039]	0.418
Autumn	0.977 [0.917–1.041]	0.471	1.003 [0.942–1.069]	0.917
Winter	1.044 [0.978–1.114]	0.189	0.981 [0.920–1.047]	0.565
**Location**				
Northern Taiwan	Reference			
Middle Taiwan	0.981 [0.929–1.036]	0.490		
Southern Taiwan	0.941 [0.889–0.996]	0.035		
Eastern Taiwan	0.905 [0.815–1.004]	0.059		
Outlets islands	1.328 [0.937–1.880]	0.111		
**Urbanization level**				
1 (The highest)	1.044 [0.969–1.125]	0.255	1.136 [1.050–1.228]	0.001
2	1.077 [1.001–1.158]	0.046	1.031 [0.958–1.110]	0.413
3	0.991 [0.896–1.096]	0.857	0.852 [0.770–0.943]	0.002
4 (The lowest)	Reference		Reference	
**Level of care**				
Hospital center	0.469 [0.443–0.497]	<0.001	0.715 [0.673–0.760]	<0.001
Regional hospital	0.513 [0.487–0.541]	<0.001	0.749 [0.710–0.790]	<0.001
Local hospital	Reference		Reference	

HR, hazard ratio; CI, confidence interval; Adjusted HR, Adjusted variables listed in the table.

Table 3 presents the pregnancy factors in populations with or without previous ectopic pregnancies. Our study demonstrated that patients with EP had a significantly increased conceiving rate of 1.16-fold (95% confidence interval [CI] 1.103–1.220; *p* < 0.001) compared to those without EP. The difference in pregnancy rate between women with previous EP and women without EP was described as follows: women whose age falls between 12 and 19 years old and ≥ 30 years old, pay a lower insurance premium (< 35,000 NT$), without complications such as anemia and shock, pay a visit in all seasons except summer, regardless of their urbanization level and choice of medical facilities.

**TABLE 3:** Factors of pregnancy stratified by variables listed in the table by Cox regression.

	With ectopic pregnancy			Without ectopic pregnancy			With *vs.* without ectopic pregnancy		
Stratified	Event	PYs	Rate (per 10^5^ PYs)	Event	PYs	Rate (per 10^5^ PYs)	Ratio	Adjusted HR [95% CI]	*P-Value*
**Total**	2,089	49,353.69	4,232.71	5,602	178,041.29	3,146.46	1.345	1.160 [1.103–1.220]	<0.001
**Age group (years)**									
12–19	41	422.70	9,699.49	56	1,149.21	4,872.93	1.990	1.798 [1.176–2.750]	0.007
20–29	1,033	9,903.66	10,430.48	3,155	32,214.43	9,793.75	1.065	1.028 [0.957–1.103]	0.450
30–39	982	19,797.57	4,960.21	2,322	62,651.24	3,706.23	1.338	1.303 [1.209–1.405]	<0.001
≧40	33	19,229.76	171.61	69	82,026.41	84.12	2.040	1.986 [1.308–3.016]	0.001
**Insured premium (NT$)**									
<18,000	2,019	47,750.86	4,228.20	5,450	173,269.24	3,145.39	1.344	1.153 [1.095–1.214]	<0.001
18,000–34,999	49	1,206.55	4,061.15	107	3,585.47	2,984.26	1.361	1.574 [1.099–2.254]	0.013
≧35,000	21	396.28	5,299.29	45	1,186.58	3,792.43	1.397	0.937 [0.525–1.673]	0.826
**Anemia**									
Without	2,074	49,028.66	4,230.18	5,570	177,213.40	3,143.10	1.346	1.157 [1.100–1.218]	<0.001
With	15	325.03	4,615.02	32	827.89	3,865.24	1.194	1.539 [0.703–3.369]	0.281
**Shock**									
Without	2,089	49,151.33	4,250.14	5,602	177,050.14	3,164.08	1.343	1.160 [1.103–1.220]	<0.001
With	0	202.36	0.00	0	991.15	0.00	–	–	–
**Season**									
Spring	463	11,424.42	4,052.72	1,301	40,184.78	3,237.54	1.252	1.117 [1.004–1.243]	0.043
Summer	498	11,961.81	4,163.25	1,405	46,093.50	3,048.15	1.366	1.086 [0.980–1.204]	0.117
Autumn	595	13,710.05	4,339.88	1,517	50,167.17	3,023.89	1.435	1.241 [1.128–1.366]	<0.001
Winter	533	12,257.42	4,348.39	1,379	41,595.84	3,315.24	1.312	1.187 [1.073–1.314]	0.001
**Urbanization level**									
1 (The highest)	725	18,016.60	4,024.07	1,957	61,005.16	3,207.93	1.254	1.096 [1.008–1.195]	0.035
2	950	21,881.68	4,341.53	2,492	77,532.01	3,214.16	1.351	1.181 [1.095–1.273]	<0.001
3	173	4,374.48	3,954.76	469	15,865.11	2,956.17	1.338	1.232 [1.033–1.470]	0.020
4 (The lowest)	241	5,080.93	4,743.23	684	23,639.00	2,893.52	1.639	1.196 [1.031–1.388]	0.019
**Level of care**									
Hospital center	428	15,301.51	2,797.11	1,277	57,203.35	2,232.39	1.253	1.106 [0.991–1.235]	0.072
Regional hospital	568	17,620.83	3,223.46	1,555	65,240.51	2,383.49	1.352	1.172 [1.064–1.291]	0.001
Local hospital	1,093	16,431.35	6,651.92	2,770	55,597.43	4,982.24	1.335	1.192 [1.111–1.280]	<0.001

PYs, Person-years; Adjusted HR, Adjusted Hazard ratio: Adjusted for the variables listed in Table 2. CI, confidence interval.

To assess the long-term trends, a 14-year follow-up was conducted using Cox regression analysis, as shown in Table 4. Adjusted hazard ratio (HR) of pregnancy were recalculated each year. In the first 5 years, the adjusted HR in the EP group was lower than that in the control group, ranging from 0.360 to 0.921-fold. However, when tracked for a longer duration, the fecundability of EP patients increased over time. They had a much higher adjusted HR of pregnancy, and the value reached a significant difference between the 10^th^ and 14^th^ years, which is 1.075–1.16 more than that of the control group.

**TABLE 4:** Adjusted HR of pregnancy by using Cox regression among different tracking years.

Ectopic pregnancy (with *vs.* without)	Adjusted HR [95% CI]	*P-Value*
**In the tracking of X year(s)**		
1	0.360 [0.317–0.409]	<0.001
2	0.674 [0.627–0.724]	<0.001
3	0.789 [0.741–0.840]	<0.001
4	0.886 [0.816–0.918]	<0.001
5	0.921 [0.871–0.975]	0.004
6	0.975 [0.923–1.031]	0.376
7	0.983 [0.932–1.037]	0.531
8	1.020 [0.967–1.076]	0.463
9	1.042 [0.989–1.099]	0.124
10	1.075 [1.021–1.132]	0.006
11	1.113 [1.057–1.172]	<0.001
12	1.129 [1.072–1.198]	<0.001
13	1.148 [1.091–1.208]	<0.001
14 (Total)	1.160 [1.103–1.220]	<0.001

HR, hazard ratio; CI, confidence interval, Adjusted HR: Adjusted variables listed in Table 2.

## 4 Discussion

The investigation of the epidemiology of EP is particularly significant in Taiwan. Previous studies have found that the intrauterine second pregnancy rate was lower in women whose first pregnancy was an EP or intrauterine pregnancy (40.5% vs. 54.5%) (10). This study is the first to specifically focus on future pregnancy rates among nulligravida with and without a history of EP. We extracted data from the large NHIRD database to ensure a robust and representative sample size. The 14 years of longitudinal analysis provided a comprehensive understanding of changes in fecundability over time after EP. Various factors, such as age, anemia, and income level, were studied to provide comprehensive insights and enhance the reliability and significance of the research.

In this study, women in the EP group had a cumulative pregnancy rate of 41.55% and a 1.16 times higher intrauterine pregnancy than those in the group without EP. These findings are consistent with those of a Canadian study that reported a similar pregnancy rate (40.5%) after EP (10). In contrast, a study in Turkey reported a notably higher pregnancy rate after EP, reaching 83.2% (84/101) (11). These results suggest that women with a history of EP may have greater potential for childbirth. In the current study, we analyzed 14 years of data to describe these phenomena in more detail. Statistics demonstrated that the annual pregnancy rate of patients with EP was initially lower and surpassed that of the control group at the 9^th^ year. This might be because women with EP may have recently undergo surgery such as salpingectomy, or medical treatment such as methotrexate (MTX), which may have a great physical and psychological impact. A previous study revealed that 3.64% of patients with EP required repeated surgeries for recurrent ectopic pregnancies (12). One study found that women with a history of EP had a lower delivery rate (69%) compared to women without (13). These results indicated that fertility might be compromised by having a history of EP (13). However, our study revealed the pregnancy ability of EP patients increased over time. Furthermore, Kårhus et al. (14) revealed the daughters of mothers with a history of EP had a 1.5-fold (95% CI 1.2–1.9) risk of EP. This may be the result of similar lifestyles of mothers and daughters, such as cigarette smoking, alcohol consumption, and sexual and contraceptive choices.

In our study, we analyzed the age range of 12–50 years, considering that most women start developing secondary sexual characteristics during their teenage years and may face postmenopausal issues after the age of 45. We noted that the majority of EP cases occurred in women older than 30 years (65.51%); these findings align with those of a study conducted by Chouinard et al. (10). Advanced age is also a risk factor for EP (3). As the age of women attempting their first pregnancy increases, the incidence of EP also increases (13). Among all patients, those aged 20–29 years had the highest likelihood of pregnancy (adjusted HR 1.589, 95% CI 1.299–1.945; *p* < 0.001). However, when comparing the two groups, patients with EP only exhibited a superior pregnancy rate between 12 and 19 years of age (adjusted HR 1.798, 95% CI 1.176–2.750; *p* = 0.007), 30–39 years (adjusted HR 1.303, 95% CI 1.209–1.405; *p* < 0.001), and ≥ 45 years (adjusted HR 1.986, 95% CI 1.308–3.016; *p* = 0.001). Although there was no statistical significance when comparing the two 20–29 year-old groups, both the EP and control groups demonstrated the highest pregnancy rates in the age range of 20–29 years.

Additionally, our study revealed that anemia was more prevalent among patients with EP, but the incidence of shock was not significant between two groups. This might be the result of proper and timely treatment. Mothers with EP who did not experience complications such as anemia and shock exhibited higher pregnancy rates than those without a history of EP.

Analyzing the residential area of patients with EP provides the government with insights into subsidies should be allocated and helps researchers and the private sector identify target populations. For example, the majority of patients EP are from Northern Taiwan, a more urbanized area (urbanization levels 1 and 2) and economically disadvantaged area. The distribution of urbanization levels and economic status was similar to that in a previous study (10). The EP relapse rate is 10% and increases to 25% when women with more than one EP history (3). Some preventive measures or medical care can be enforced in these areas to reduce the medical costs and alleviate the pain experienced by mothers with EP.

Our study showed that patients with EP were more likely to be treated in local hospitals than those without (40.27% vs. 36.79%). Regarding pregnancy rates, local hospitals scored the highest. This might be attributed to the referral of difficult cases to hospital centers.

Furthermore, our study highlighted that a higher-income population has a much higher pregnancy rate. However, when we consider EP, a higher pregnancy rate was found in women who paid less than 35,000 NT. This may be because the assisted reproductive technology (ART) is more affordable in the higher income group. Although the NHI provides a wide variety of medical care services equally to every citizen, regardless of socioeconomic differences, the in vitro fertilization (IVF) technique was not included in our study. The average cost of IVF was estimated to be 3,817 USD per cycle in 2002 (15), which was 32% of Taiwan’s per capita income in the same year (11,914 USD) (16). In July 2021, the government began to subside 3,333 USD for IVF treatment. With improvements in IVF techniques and treatment affordability, the role of ART in EP has become increasingly significant. A study conducted by Xue et al. demonstrated that there was no difference in pregnancy, live birth, miscarriage, or ectopic pregnancy rates in patients undergoing IVF with respect to EP history (17). Similarly, Cai et al. (18) revealed that when women undergoing IVF, there was no statistically significant differences in live birth, clinical pregnancy, miscarriage and EP rates between patients with EP and intrauterine pregnancy or those who had never conceived (18).

This study had various limitations. We did not compare the pregnancy condition of patients with EP according to the treatment they received, such as observation, medical treatment with MTX, surgical treatment, or surgery for failed MTX treatment. However, recent studies have demonstrated that the ability to achieve a new pregnancy, time to a new pregnancy, and prognosis of pregnancy are not associated with treatment methods (11, 19–22). This change may be caused by advancements in the diagnosis of EP, improvements in surgical skills, and better ART techniques. Additionally, neonatal outcomes were not assessed in our study. A population-based retrospective cohort study of 1,117,571 pregnant women in Canada revealed that intrauterine pregnancy after EP had 1.27 times the risk of preterm birth (95% CI 1.18–1.37), 1.20 times the risk of low birth weight (95% CI 1.10–1.31), 1.21 times the risk of placental abruption (95% CI 1.04–1.41), and 1.45 times the risk of placenta previa (95% CI 1.10–1.91) compared to women whose first pregnancy was intrauterine (10). This indicates that close monitoring during the prenatal stage benefits both mothers and neonates. Furthermore, we could not distinguish between the willingness to have a child in our study. One study demonstrated that 208 of 298 patients (67.7%) with a history of EP who attempted to conceive became pregnant, a higher number of patients than that in our study (21). Although the pregnancy rate increased after excluding women who did not desire offspring, this did not influence the results of the comparison between the study and control groups. Lastly, the current study did not consider marital status, sexual preference, or infertility (in both males and females). Further studies are needed to understand the influence of these factors on the outcomes.

## 5 Conclusion

This study revealed that patients with ectopic pregnancies have distinct future pregnancy rates. This study analyzed various variables in patients with EP to provide insight into the socioeconomic background of these populations, including their places of residence, tendencies to seek medical help, income levels, and potential complications. Subsequently, we associated these variables with pregnancy rates through intra- and intergroup comparisons. The findings revealed that, over time, patients with a history of EP showed an improved pregnancy rate, particularly noticeable 10 years after the initial event.

## Data Availability

The raw data supporting the conclusions of this article will be made available by the authors, without undue reservation.
